# The impact of emotional distress on response to radiofrequency ablation

**DOI:** 10.1097/PR9.0000000000001253

**Published:** 2025-04-03

**Authors:** Andrea Nikolis, Louis Nikolis, Zan Wynia, Carlos Murillo, Jason Friedrich, Yoni K. Ashar

**Affiliations:** a Department of Physical Medicine & Rehabilitation, University of Colorado Anschutz Medical Campus, Aurora, CO, USA; b Division of Internal Medicine, University of Colorado Anschutz Medical Campus, Aurora, CO, USA

**Keywords:** Pain, Chronic pain, Interventional pain, Radiofrequency ablation, Emotional distress, Depression, Anxiety, Biopsychosocial approach, Biopsychosocial pain management

## Abstract

**Introduction:**

Radiofrequency ablation (RFA) is commonly performed for facet-mediated cervical, thoracic, and lumbar spine pain. Inadequate relief from RFA may be because of biopsychosocial factors not addressed with RFA.

**Objectives:**

To investigate the effects of baseline emotional distress on RFA treatment response.

**Methods:**

Patients who completed self-reported surveys, Patient-Reported Outcomes Measurement System (PROMIS), from January 2021 through July 2023 were included. Additional baseline information was obtained by retrospective review. The primary outcome was PROMIS Pain Intensity T-scores at 6 weeks, 3 months, and 6 months after RFA. The main predictor of interest was emotional distress, computed as average baseline PROMIS T-scores for depression and anxiety. Interaction terms tested how the influence of baseline emotional distress and pain intensity changed over the follow-up period.

**Results:**

Data were available for 154, 101, 91, and 83 participants at baseline, 6 weeks, 3 months, and 6 months post-RFA, respectively. Baseline emotional distress (*P* = 0.04) and baseline pain intensity (*P* = 0.01) were both associated with worse pain intensity at follow-up, controlling for each other. Interaction terms were not significant, though visual inspection of trends suggested that the influence of baseline emotional distress may increase as the follow-up period lengthens.

**Conclusion:**

Patients reporting higher levels of baseline emotional distress experience less pain reduction after RFA, controlling for baseline pain intensity. Patients with greater emotional distress may benefit from treatments addressing emotional distress, in addition to or instead of RFA.

## Abstract 2

Patients with higher baseline depression and anxiety (emotional distress) report less reductions in neck and back pain intensity after radiofrequency ablation.

## 1. Introduction

Chronic neck and back pain are leading causes of disability and health care costs in the United States.^22^ From a mechanical perspective, axial pain in the cervical, thoracic, and lumbar spine may arise from the zygapophysial joints, also known as facet joints. Per the available literature, the most studied intervention to treat confirmed facet joint pain is medial branch radiofrequency neurotomy, also known as radiofrequency ablation (RFA).^5^ In a national database study conducted from 2007 to 2016, the incidence of lumbar RFA increased by 9.7% annually (130.6% increase over the study period), and the procedure cost increased by 2.3% annually.^17^ However, RFA does not account for the biopsychosocial, multifactorial contributions to chronic pain, which contribute to maladaptive behaviors and physiologic changes in the central nervous system that can be associated with resistance to peripherally focused treatments.^2^ Several biopsychosocial factors, including high levels of emotional distress, disability, somatization, and pain catastrophizing, are associated with poorer prognosis in low back pain.^4,6,14,15^ This may help explain why RFA, on average, has a small, short-term effect at best relative to sham RFA procedures.^3,19^

Emotional distress, such as depression and anxiety, is often associated with chronic pain, yet it remains unclear how emotional distress may affect RFA response.^2,18^ Overall, emotional distress is associated with increased pain chronicity, increased pain intensity, increased disability, and overall worse general health.^6,8,12,15^ This study aimed to evaluate the predictive value of pre-procedural emotional distress on RFA response. We tested the effects of RFA on pain at 3 different follow-up time points, to investigate whether the predictors of RFA response changed over the course of the follow-up period.

## 2. Methods

This retrospective study was exempt by the Institutional Review Board through the study institution. Patients with neck and back pain who underwent cervical, thoracic, or lumbar RFA between January 2021 and July 2023 at University of Colorado Health Outpatient Spine Clinics were included for retrospective review. Standard procedural guidelines for conventional RFA were used. Participants were required to undergo 2 diagnostic medial branch blocks with a required minimum of 80% pain relief after each block to proceed with RFA, in which the target was heated to 80°C for 60 to 90 seconds. Inclusion criteria were individuals 18 years of age and older who underwent RFA during this period and who completed a self-report survey before the RFA. Exclusion criteria were those who did not undergo RFA and those who did not complete a baseline survey before the RFA. All participants were invited to complete follow-up surveys at 6 weeks, 3 months, and 6 months post-RFA. Participants were required to have data at a given time point to be included in analyses for that time point. The first available patient entry was included for patients who had multiple RFA procedures during the data collection period, and later entries for these patients were excluded. For each patient, electronic medical records were retrospectively reviewed for baseline demographic information and the level and laterality of the RFA.

The primary outcome of this study was Patient-Reported Outcomes Measurement System (PROMIS) Pain Intensity Short Form T-scores at 6 weeks post-RFA. To evaluate if change in pain scores from baseline were significant at each follow-up time point, 1-sample pre–post *t* tests were conducted. To evaluate predictors of RFA response, a linear mixed model was estimated. The main predictor of interest was emotional distress, computed by averaging pretreatment PROMIS T-scores from PROMIS Depression Computer Adaptive Test (CAT) and PROMIS Anxiety CAT (which were correlated at *r* = 0.78). Patient-Reported Outcomes Measurement System T-scores are calculated with respect to a reference population representative of a broad US population sample, with mean = 50 and a SD = 10. Outlier emotional distress observations were discovered through visual inspection of scatter plots, and a single outlier was removed. Other candidate predictors were baseline pain, age, body mass index, sex, prior RFA, RFA location (lumbar or cervical/thoracic), and PROMIS Sleep Disturbance CAT T-score. Continuous predictors were mean centered before model fitting. The mixed effect model included all available data, with time point coded as a numeric variable (0, 1, and 2 for 6 weeks, 3 months, and 6 months, respectively), a random intercept for each subject, and interaction terms for time point × baseline pain and time point × baseline emotional distress.

## 3. Results

**Figure 1.**
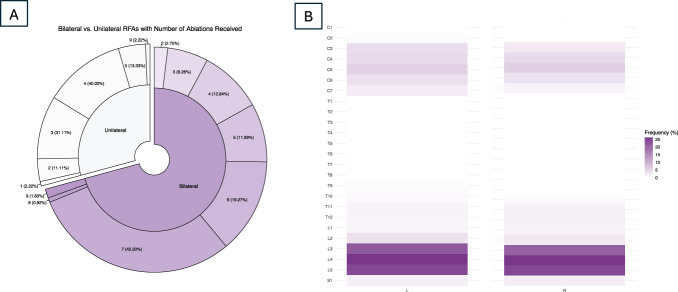
Distribution and frequency of RFAs performed. (A) Percentage of RFAs that were unilateral vs bilateral (inner circle) and the number (percentage) of medial branches targeted with each procedure (outer circle). (B) Frequency with which each medial branch was targeted. C, cervical; T, thoracic; L, lumbar; S, sacral; L, left; R, right. RFA, radiofrequency ablation.

At baseline, data were available for 154 participants (46.8% women; mean age 68 ± SD years, as seen in Table 1), with 101 observations included at 6 weeks post-RFA, 91 observations at 3 months post-RFA, and 83 observations at 6 months post-RFA. The majority of RFA procedures were done bilaterally in the lumbar spine, with the most common procedure levels involving the L3, L4, and L5 medial branches/dorsal rami, illustrated in Figure 1. The sample had an average age of 67.98 years (SD = 12.68), was 53.2% male, and was 93.5% white. Mean pain at all time points and baseline anxiety, depression, and emotional distress are provided in Table 1. Examining participants with data at each follow-up time point, there was a mean reduction from baseline of 4.51 points at 6 weeks (SD of change scores = 6.64, *P* < 0.01), of 3.74 points (SD = 5.80, *P* < 0.01) at 3 months, and of 3.4 points (SD = 7.23, *P* < 0.01) at 6 months.

**Table 1:** Demographics and patient-reported outcomes.

No. of subjects	154
Age (y)	
Mean (SD)	68.0 (12.7)
Median [min, max]	70.0 [30.0, 90.0]
BMI (kg/m^2^)	
Mean (SD)	27.6 (5.19)
Median [min, max]	26.8 [17.4, 45.7]
Sex	
Female	72 (46.8%)
Male	82 (53.2%)
Race or ethnicity	
White	144 (93.5%)
Asian	4 (2.6%)
Hispanic	3 (1.9%)
Other	2 (1.3%)
Declined to answer	1 (0.6%)
Marital status	
Married	96 (62.3%)
Divorced/separated	19 (12.3%)
Single	18 (11.7%)
Domestic partner	3 (1.9%)
Widowed	6 (3.9%)
Unknown	10 (6.5%)
Other	2 (1.3%)
Location of RFA procedure	
Cervical/thoracic	38 (24.7%)
Lumbar	116 (75.3%)
Baseline anxiety PROMIS T-score	
Mean (SD)	54.3 (9.39)
Median [min, max]	54.2 [32.9, 84.9]
Missing	3 (1.9%)
Baseline depression PROMIS T-score	
Mean (SD)	49.1 (9.61)
Median [min, max]	49.5 [34.2, 84.4]
Missing	4 (2.6%)
Baseline pain intensity PROMIS T-score	
Mean (SD)	54.5 (4.75)
Median [min, max]	54.5 [40.2, 71.8]
Missing	15 (9.7%)
6-wk pain intensity PROMIS T-score	
Mean (SD)	49.8 (6.48)
Median [min, max]	49.4 [30.7, 67.4]
Missing	40 (25.9%)
3-mo pain intensity PROMIS T-score	
Mean (SD)	50.9 (5.58)
Median [min, max]	52.1 [40.2, 67.4]
Missing	50 (32.5%)
6-mo pain intensity PROMIS T-score	
Mean (SD)	50.8 (6.50)
Median [min, sax]	52.1 [30.7, 67.4]
Missing	58 (37.7%)

Race and ethnicity were asked as 1 question (“Please indicate your ethnicity”), and participants were asked to select the category that best describes them. PROMIS T-scores are standardized scores with a mean of 50 and a SD of 10, with the reference population corresponding to the general US population.

BMI, body mass index; PROMIS, Patient-Reported Outcomes Measurement System; RFA, radiofrequency ablation.

**Figure 2.**
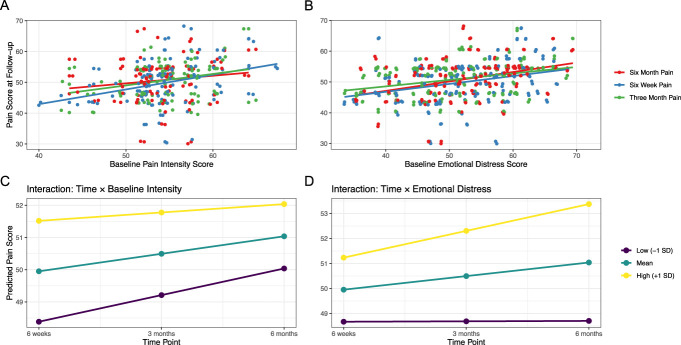
Effects of baseline pain and emotional distress on follow-up pain intensity. (A) Relationship between baseline pain and pain intensity at different follow-up time points. The association is the strongest at 6 weeks post-RFA. (B) Relationship between baseline emotional distress and pain intensity at different follow-up time points. Relationship is the strongest at 6 months post-RFA. Interactions of (C) time × baseline pain and (D) time × emotional distress, suggesting that as the follow-up time lengthens, the influence of baseline emotional distress increases and the influence of baseline pain intensity decreases, although interaction terms were not significant with this sample size. RFA, radiofrequency ablation.

Among the covariates, only baseline pain (β = 0.35, *P* = 0.01) and emotional distress (β = 0.15, *P* = 0.04) were predictive of follow-up pain intensity. Neither interaction term testing how the effects of baseline pain and emotional distress varied over the follow-up time period was significant. Visual inspection of these interactions suggested that as the follow-up period lengthens, the influence of baseline emotional distress may increase and the influence of baseline pain intensity may decrease (Fig. 2).

## 4. Discussion

Radiofrequency ablation focuses on peripheral physiology and does not target other behavioral and psychological contributors to pain, such as emotional distress. Emotional distress, including depression and anxiety, may contribute to physiologic adaptations of the central nervous system that are associated with central sensitization and chronic pain.^2^ Our findings demonstrate that RFA is less effective at reducing pain intensity in the presence of emotional distress.

Previous studies have suggested that greater baseline functional impairment, baseline pain scores, pain chronicity, and obesity may be associated with worse outcomes after RFA.^9,10,13^ To date, only one prior study of 41 patients found that depression is associated with poorer RFA outcomes. However, this study was unable to adequately assess depression as a predictor of long-term RFA success, given its small sample size and loss of follow-up data.^18^

The association of emotional distress on pain and interventional success has been better studied in lumbar spine surgery. Higher levels of baseline depression are associated with worse outcomes after lumbar spinal fusion.^23^ When compared with patients without pre-existing depression and anxiety, those with depression and anxiety have worse outcomes after spine surgery, including ongoing pain and functional impairment.^16^

Our findings extend the current evidence regarding the relationship of emotional distress on RFA outcomes. In patients with greater emotional distress, nociplastic processes (which are not directly targeted by RFA) may be a central contributor to ongoing pain. Our findings support the centrality of emotional distress in chronic pain frameworks centering fear and avoidance and negative affective learning.^1,21^

As RFA does not target the central nervous system, clinical discretion must be used when recommending this treatment for patients with centralized symptoms. Implications for clinical practice include carefully selecting patients for RFA by screening for emotional distress, providing patient counseling about the impact of emotional distress on RFA response, and considering therapies that target emotional distress, such as psychotherapy, either instead of or alongside RFA.

Neither interaction term in our model was statistically significant, potentially because of our medium-sized sample. At the same time, visual inspection of interaction plots showed a growing relationship between higher baseline emotional distress and pain as the follow-up period lengthened. This suggests that emotional distress may be especially important for long-term patient outcomes and that providing treatments addressing emotional distress is especially important for long-term pain reduction.

An additional point to consider is the influence of the placebo effect. Randomized trials comparing RFA to sham procedures have varied results, with most larger studies reporting no statistically significant differences between real and sham RFA groups, along with substantial improvement in pain scores after sham treatments.^19,20^ This suggests that a large portion of the benefit of RFA is because of the placebo effect. As there is some evidence that placebo treatments are less effective in patients with higher depression and anxiety,^7,11^ one explanation of our findings is that patients with greater emotional distress are less likely to benefit from placebo elements of RFA. To directly investigate this, future studies are needed to test whether baseline emotional distress predicts response to sham RFA (eg, in control arms of randomized RFA trials).

Limitations of this study include the retrospective nature, moderate sample size, and loss of follow-up data. In conclusion, our findings underscore the importance of screening and managing biopsychological patient characteristics to provide personalized care and improve treatment success. Patients reporting higher levels of baseline emotional distress are less likely to experience a pain reduction following RFA, irrespective of baseline pain intensity.

## Disclosures

Y. K. Ashar is a consultant with Lin Health and the Pain Reprocessing Therapy Center. The remaining authors have no conflicts of interest to declare. 
